# Traumatic brain injury, nutritional status and outcomes: a registry based cohort study

**DOI:** 10.1186/2197-425X-3-S1-A437

**Published:** 2015-10-01

**Authors:** A Peetz, KM Mogensen, JD Rawn, A Salim, KB Christopher

**Affiliations:** Division of Trauma, Burn, and Surgical Critical Care, Brigham and Women's Hospital, Boston, MA USA; Department of Nutrition, Brigham and Women's Hospital, Boston, MA USA; Division of Cardiac Surgery, Brigham and Women's Hospital, Boston, MA USA; Renal Division, Brigham and Women's Hospital, Boston, MA USA

## Introduction

Severe Traumatic Brain Injury (TBI) is associated with hypermetabolism and increased protein catabolism. While studies suggest that nutritional support in TBI may be beneficial, to date, limited information exists regarding the association between existing malnutrition at ICU admission and patient outcomes.

## Objectives

We hypothesized that malnutrition would be associated with increased risk of short term mortality.

## Methods

We performed a single center observational study of patients treated in medical and surgical intensive care units in Boston, Massachusetts. We studied 1,685 patients age ≥ 18 years, who received critical care following TBI between 1997 and 2011. The exposure of interest, malnutrition, was determined via a Registered Dietitian formal assessment within 48 hours of ICU admission and categorized as non-specific malnutrition, protein-energy malnutrition, at risk of malnutrition or well-nourished and determined by data related to anthropometric measurements, clinical signs of malnutrition, malnutrition risk factors, and metabolic stress. The primary outcome was all cause 90 day mortality determined by the US Social Security Death Master File. Adjusted odds ratios were estimated by multivariable logistic regression models.

## Results

The cohort was 60% male, 78% white and had a mean age of 61.9 years. 5% of the cohort had sepsis, 14% had acute kidney injury and 21% had non-cardiac acute respiratory failure. 20% underwent a craniotomy. 11% of the cohort had an unplanned 30 day hospital readmission. The 30, 90 and 365-day mortality was 17.7 and 21.8 and 27.9%. In a logistic regression model adjusted for gender, ICD-9 based injury severity score (ICISS), craniotomy status and an ICU risk prediction score (inclusive of age, gender, race, Deyo-Charlson index, acute organ failure, and sepsis), patients with malnutrition (non-specific malnutrition or protein-energy malnutrition) have a 1.7 fold increased odds of 30-day mortality [adjusted OR 1.73 (95%CI 1.31-2.29), *P* < 0.001] and a 1.9 fold increased odds of 90-day mortality [adjusted OR 1.86 (95%CI 1.43-2.42), *P* < 0.001] compared to patients without malnutrition. The primary model showed good discrimination for 90-day mortality (AUC= 0.74 (95%CI 0.71- 0.77)). Further, in survivors of hospitalization (n = 1,424) patients with malnutrition at ICU admission have a 50% increased odds of 90-day post discharge mortality [adjusted OR 1.52 (95%CI 0.99-2.37), *P* = 0.059] and a 40% increased odds of 365-day post discharge mortality [adjusted OR 1.45 (95%CI 1.02-2.05), *P* = 0.035] compared to patients without malnutrition.

## Conclusions

In patients with TBI who require critical care, nutritional status is predictive of adverse outcomes. Compared to well-nourished patients, those with evidence of malnutrition have increased short term mortality. In survivors of hospitalization, malnourished patients have increases in mortality up to a year following discharge.

